# A Case Report of *Histoplasma*-Associated Empyema Treated With Intravenous Injection and Local Thoracic Irrigation of Amphotericin B Plus Medical Thoracoscopy

**DOI:** 10.3389/fpubh.2022.914529

**Published:** 2022-07-06

**Authors:** Ning Cui, Lijun Wang, Jingming Zhao

**Affiliations:** Department of Respiratory and Critical Care Medicine, The Affiliated Hospital of Qingdao University, Qingdao, China

**Keywords:** *Histoplasma*, empyema, amphotericin B, medical thoracoscopy, thoracic irrigation

## Abstract

Cases of empyema associated with *Histoplasma* infection are rarely reported. Here, we discuss a case of *Histoplasma*-associated empyema successfully treated with amphotericin B intravenous and pleural infusion therapy and multiple medical thoracoscopies. A 57-year-old Chinese woman with preexisting diabetes mellitus and gastric cancer had massive left-sided pleural effusion diagnosed by chest computed tomography. Her pleural effusion was purulent through pleural catheter drainage, and the culture of the pleural fluid showed *Escherichia coli* and Streptococcus constellation. Histopathology of the thoracoscopic pleural biopsy after hexamine silver and PAS staining supported *Histoplasma* infection. The patient was treated with intravenous injection and local thoracic irrigation of amphotericin B and continuous oral administration of itraconazole. At the same time, the patient received thoracic cannulation, daily thoracic lavage and thoracoscopy for purulent and necrotic tissue removal three times during hospitalization. The patient's pleural effusion and necrotic tissue in the pleural cavity were significantly reduced in a short time, and the clinical symptoms were significantly improved. After discharge, the patient recovered well and had no obvious complications or sequelae. Intravenous injection and local thoracic irrigation of amphotericin B are safe and effective drug therapies to treat fungal-associated empyema such as *Histoplasma*. Medical thoracoscopy effectively shortens the recovery time of empyema, improving the prognosis and reducing complications.

## Introduction

Histoplasmosis is a primary fungal disease and a granulomatous disease caused by *Histoplasma* capsulatum. Although distributed worldwide, it is most prevalent in South, Central, and North America (1).

Most *Histoplasma* infections are asymptomatic in normal immune hosts and do not result in long-term adverse sequelae. The remaining infected individuals develop one of several different clinical syndromes. A classification scheme subdivides symptomatic disease into acute pulmonary histoplasmosis, disseminated histoplasmosis, chronic pulmonary histoplasmosis, and complications from an excessive fibrotic response to the body (2). Disseminated histoplasmosis occurs primarily in immunocompromised persons, elderly individuals, and patients with underlying chronic diseases (3).

Few reports have investigated pleural effusion with *Histoplasma*, and a small number of acute histoplasmosis cases lead to pleural effusion (4, 5). Herein, we report for the first time, to our best knowledge, a case of empyema due to *Histoplasma* and bacterial infections in a diabetic patient after gastric cancer surgery treated with oral anticancer agents. We also review the literature to highlight the clinical features of this uncommon manifestation of histoplasmosis.

## Case Report

A 57-year-old female patient was admitted to the Department of Respiratory and Critical Care Medicine at our hospital on August 27, 2020. Her chief complaint was “left side chest pain for 3 months, aggravated for 1 week.” The patient had a history of diabetes for more than 10 years, and her blood sugar was well-controlled by oral hypoglycemic drugs. In May 2020, the patient had undergone surgery for a gastric antral malignant tumor and treatment with gimeracil and oteracil potassium capsules after gastric surgery.

**Figure 1:**
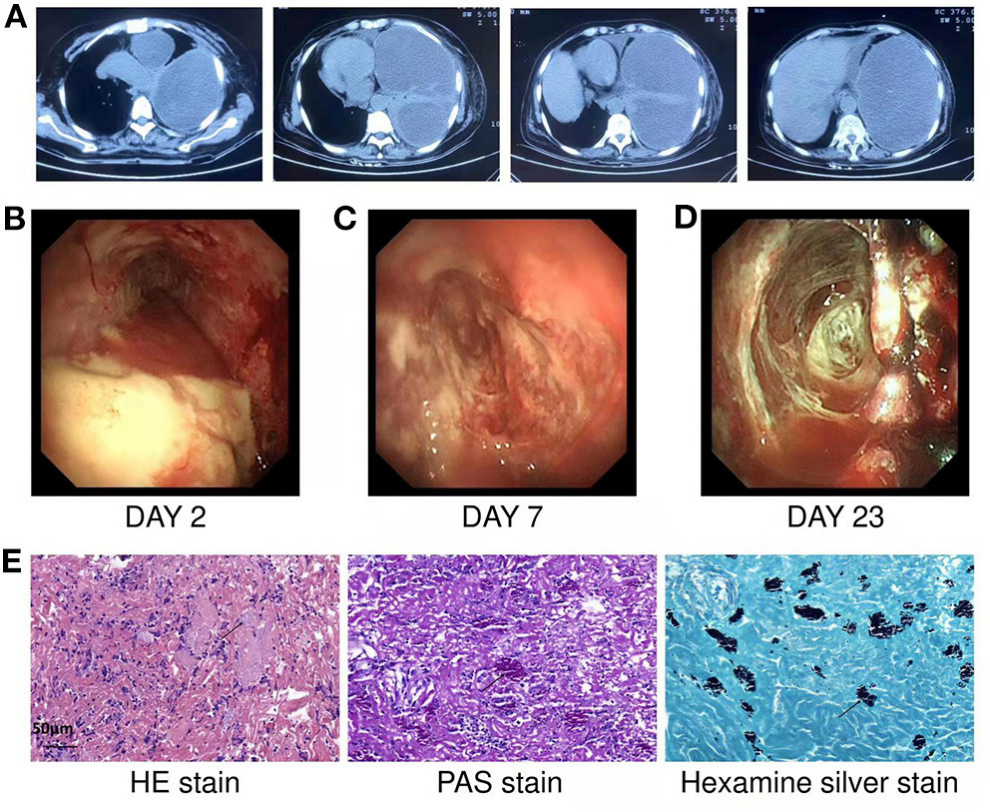
**(A)** Chest CT showed left pleural effusion with left atelectasis before admission. **(B)** On the second day of hospitalization, medical thoracoscopy revealed extensive purulent attachment to the parietal pleura, visceral pleura, and diaphragmatic pleura. **(C,D)** Show the findings of thoracoscopy on the 7th and 23rd days of hospitalization, respectively. After systemic medication, thoracic irrigation and thoracoscopy to clear necrosis and adhesions, the necrosis in the thoracic cavity gradually decreased, the pleural thickening and adhesion gradually decreased. **(E)** HE staining showed that chronic inflammatory cells infiltrated and aggregated tissue cells in the fibrous connective tissue. The cytoplasm of tissue cells was rich in powdery staining, and there were clustered, oval and light blue fungal spores (black arrows). PAS staining showed spore capsules in pink (black arrows). Silver staining showed spore capsules in black (black arrows) (Scale bar = 50 μm).

The patient presented with left-sided chest pain after gastric surgery 3 months ago. One week before admission, the patient's chest pain was aggravated, accompanied by fever, with a maximum temperature of 40°C. The patient did not respond well to the anti-infective treatment in the local hospital. The patient's blood routine data at the local hospital were as follows: white blood cell count: 13.58^*^10^9^/L; neutrophil count: 11.88^*^10^9^/L. Her chest computed tomography (CT) showed left pleural effusion with left atelectasis. There was no enlargement of hilar, no enlarged or calcified mediastinal lymph nodes, no widening of mediastinum and no compression of trachea and large blood vessels (Figure 1A). Next, the patient visited to the emergency department of our hospital, a thoracentesis catheter was inserted for drainage, and the drainage fluid was yellow and purulent. The bacterial culture results of the pleural effusion were positive for Escherichia coli and Streptococcus constellation.

On admission, the patient had a wheezing face, her left rib cage was full, and her intercostal space widened. The patient's left thoracic cavity was solid to percussion, and the left breath sounds disappeared.

The patient's admission diagnosis was purulent pleurisy, postoperative antral malignancy and diabetes mellitus type 2.

The patient's abnormal auxiliary examination results after admission were as follows: hemoglobin: 88 g/L; specific mycobacterium tuberculosis cellular immunoassay (T-SPOT.TB): positive; procalcitonin: 0.11 ng/ml; serum albumin: 22.1 g/l; ESR 44: mm/1 h.

The medical thoracoscopy was performed on the second day after admission. Extensive purulent attachments to the parietal pleura, visceral pleura, and diaphragmatic pleura were observed (Figure 1B). Multiple tissue biopsies were performed during the operation, and the purulent, necrotic tissue and adhesions in the thoracic cavity were partially removed. Postoperatively, the drainage fluid was removed from the patient using a thoracic tube. Routine examination of the pleural effusion showed that it was a grayish-yellow viscous liquid comprising primarily multinucleated cells. The Rivalta test of pleural effusion was positive.

Pleural fluid culture was still positive for *Escherichia coli* and Streptococcus constellation. The pleural fluid was negative for tuberculosis culture and acid-fast staining, meanwhile, her pleural biopsy tissue was were negative for tuberculosis culture. The lactate dehydrogenase level in the pleural effusion was 8,473 U/L, the carcinoembryonic antigen level was 0.73 ng/ml, and the adenosine deaminase level was 232 U/L. A pathological smear of the pleural effusion showed many neutrophils and a few lymphocytes and plasma cells. The pathological findings of the pleural biopsy showed chronic suppurative inflammation with necrosis and inflammatory granulation tissue formation in the fibrous connective tissue. The biopsy special staining results (Figure 1E) were as follows: hexamine silver (+), PAS (+), acid fast (–). Fungal spores were observed on staining, and the morphology supported *Histoplasma*. And no malignant cells were found in pleural fluid and pleural biopsy.

The patient was diagnosed with empyema, and the pathogens were *Escherichia coli*, Streptococcus constellation, and *Histoplasma*. For *Escherichia coli* and Streptococcus constellation infections, Cefoperazone sulbactam sodium combined with levofloxacin and ceftriaxone monotherapy were successively administered. *Histoplasma* infection was treated with intravenous injection of deoxycholate amphotericin B at an initial dose of 3 mg and local thoracic irrigation of deoxycholate amphotericin B at an initial dose of 5 mg. The dose of amphotericin B was increased by 5 mg daily for both intravenous injection and thoracic irrigation to a maximum dose of 25 mg. Intravenous injection was administered for 15 days, and thoracic irrigation was administered for 8 days.

During hospitalization, the patient underwent thoracoscopy 3 times. Each time the necrosis and adhesions were cleaned using biopsy forceps. The necrosis in the thoracic cavity gradually decreased, the pleural thickening and adhesion gradually decreased (Figures 1B–D), and the left lung gradually re-expanded. The histopathological special staining of the last two thoracoscopic pleural biopsies results were as follows: hexamine silver (–), PAS (–), and acid fast (–). No definite fungus was found by histopathological staining and pleural tissue culture showed no bacterial or fungal growth.

**Figure 2:**
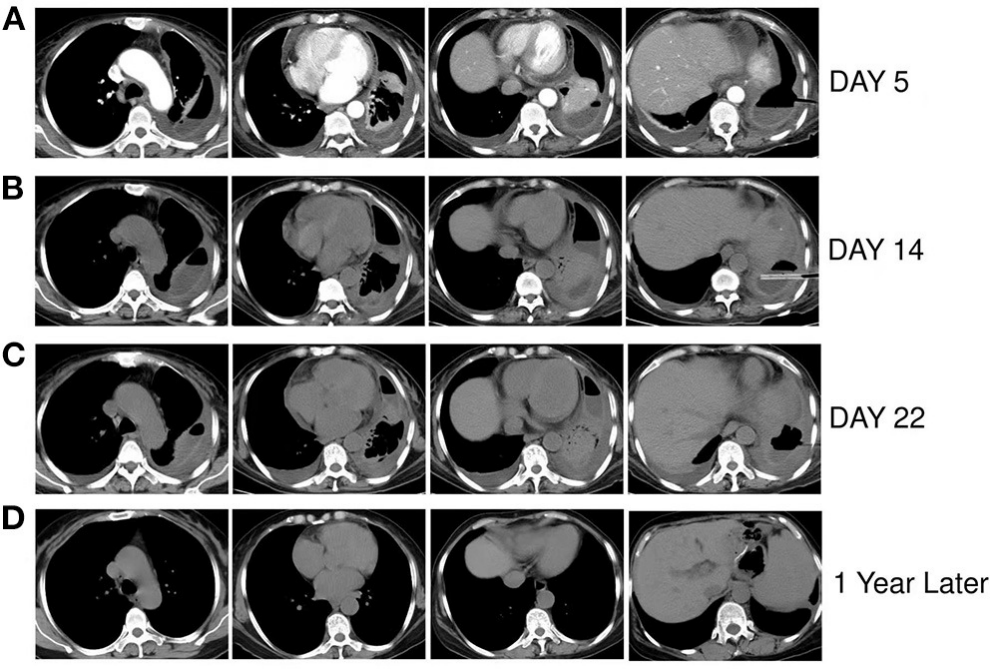
**(A–C)** Are respectively the chest CT scans of the patient on the 5th, 14th, and 22nd days of hospitalization, showing the left lung was gradually re-expanded, the left pleural effusion was gradually reduced, and the left pleural thickening was gradually decreased. **(D)** Chest CT of the patient nearly 1 year after discharge, showing that the left lung recruited, a very small amount of pleural effusion on the left side and slightly thick pleura.

The characteristics of pleural effusion drainage fluid were significantly improved; it was a pale yellow and clear liquid, and multiple cultures were negative. During hospitalization, the patient underwent three chest CT scans (Figures 2A–C), which revealed that the left lung was gradually re-expanded, the left pleural effusion was gradually reduced, and the left pleural thickening was gradually decreased. She was discharged after 34 days of hospitalization, and her symptoms had improved significantly upon discharge. She had tolerated amphotericin B treatment well; no obvious adverse reactions occurred. After discharge, the patient's anti-infection regimen was to take cefdinir 0.1 g three times a day for 10 days and itraconazole, 200 mg twice a day, for 18 days. The last re-examination of the chest CT about 1 year later, showed that the left lung was re-expanded, with little pleural effusion and pleural thickening on the left side (Figure 2D).

## Discussion

Lung infections caused by *Histoplasma* are usually asymptomatic or mildly symptomatic. The severity of infection depends primarily on the amount of the initial inoculum and immune status of the host. Pulmonary infection and extrapulmonary spread resolve spontaneously in >99% of cases as cellular immunity develops. In patients who fail to mount an effective immune response, the infection is usually progressive (6). Here, we report case of a female patient with *Histoplasma*-associated empyema. Severe infection in this patient was associated with decreased immune function due to diabetes, gastric malignancy and probable latent TB infection suggested by the positive result of T-SPOT.TB. Pleural effusion in patients with histoplasmosis is rare, with an incidence ranging from 0 to 6% (7, 8), although *Histoplasma* pneumonia usually adjoins the pleural space. Massive pleural effusion has only been reported in few cases (9, 10). No empyema associated with *Histoplasma* infection has been reported to date. The pathogenic agents in the pleural fluid of this patient were Escherichia coli, Streptococcus constellation and *Histoplasma*. Her remission was not ideal after receiving antibacterial treatment. The purulent pleural effusion gradually improved after treatment for *Histoplasma* infection. Therefore, this patient's massive purulent pleural effusion was at least partially caused by *Histoplasma* infection.

Microscopic examination of pleural fluid for histoplasmosis is usually negative (5, 11–14). Only a few studies have reported that pleural effusion caused by *Histoplasma* can be diagnosed microbiologically by pleural fluid culture (15–17). Culture of the pleural effusion in this patient also did not reveal *Histoplasma*. Histopathology remains the standard diagnostic method for *Histoplasma* infection, in addition to culture. Morphologic diagnosis of *Histoplasma* capsulatum is based on finding oval, narrow-based, distinctive 2–4-micron budding yeasts. Other organisms, such as *Blastomycesdermatitidis, Candida glabrata*, and *Leishmania*, can mimic the appearance of *Histoplasma* capsulatum. However, these similar pathogenic microorganisms are much larger, measuring 10–15 microns, and show broad-based budding (18). Tissue samples should be stained with GMS or PAS instead of hematoxylinand eosin staining (19). The diagnosis of histoplasmosis in this patient was established by pleural biopsy pathology, meeting the definition of proven invasive fungal disease of the latest recommendations by Donnelly et al. (20).

Most immunocompetent cases with mild illness of histoplasmosis require no specific treatment. Amphotericin B clearly benefits patients with disseminated, progressive disease, particularly immunocompromised patients. Guazzelli L reported in 2011 that amphotericin B lavage treatment achieved good results in 3 cases of fungal empyema (21). Amphotericin B does not easily enter the pleural cavity, and the drug concentration in the pleural cavity is low (22–25). Successful treatment of fungal empyema requires maintaining the concentration of the drug in the pleural cavity above its MIC. This patient received intravenous injection and local thoracic irrigation of amphotericin B to treat thoracic infection caused by *Histoplasma* with good efficacy and no systemic or local adverse effects. However, amphotericin B is not curative for *Histoplasma* infection. Nearly all such patients relapse after the completion of amphotericin, prompting the current recommendation for suppressive azole antifungal drug therapy following amphotericin B treatment (26, 27). The IDSA Guidelines on histoplasmosis treatment does not mention the treatment of Histoplasma-associated empyema (28). Referring to previous literature reports, this patient was administered oral itraconazole for 18 days after a full course of amphotericin B treatment; finally, her *Histoplasma* pleural infection was well-controlled. Patients with large infectious pleural effusion are indicated for pleural drainage. Previously, thoracic drainage was used to treat acute empyema however, it is ineffective and time-consuming. In recent years, medical thoracoscopy demonstrated a good effect when applied to empyema. Medical thoracoscopy can be performed by the respiratory physician under local anesthesia and spontaneous ventilation. It allows access to the pleural cavity with a thoracoscope via a small chest wall incision, allowing the physician to perform pleural biopsy with high accuracy and drain pleural effusion. Compared to Video-assisted thoracoscopic surgery (VATS), medical thoracoscopy is less invasive, has a comparable diagnostic yield, becoming an important method to treat empyema (29). This patient underwent three medical thoracoscopy to drain pleural effusion and remove necrotic tissue, which facilitated the recovery and reduced subsequent complications. Some patients with acute histoplasmosis reported may have residual pleural thickening, even extensive pleural fibrosis (13). Our patient had only mild pleural thickening and no pleural fibrosis remaining after 1 year of follow-up.

This report describes a rare case of empyema associated with *Histoplasma* with good outcome. For immunocompromised patients with pleural effusion, physicians should be vigilant regarding the presence of opportunistic infections, such as *Histoplasma* infection, since delayed recognition of histoplasmosis can complicate the course and increase mortality. Furthermore, pleural effusion microbial detection and pleural biopsy pathology should be performed. Massive pleural effusion due to *Histoplasma* infection requires intravenous injection and thoracic irrigation of amphotericin B followed by azole antifungal therapy. For empyema associated with *Histoplasma*, medical thoracoscopy is recommended for the diagnosis and treatment to facilitate diagnosis and treatment.

## Data Availability Statement

The original contributions presented in the study are included in the article/supplementary material, further inquiries can be directed to the corresponding author.

## Ethics Statement

Written informed consent was obtained from the individual(s) for the publication of any potentially identifiable images or data included in this article.

## Author Contributions

JZ and NC had the initial idea to perform this study. JZ and LW collected the clinical data. NC wrote the manuscript that was read and approved by all authors. All authors contributed to the article and approved the submitted version.

## Funding

This work was funded by the Medicine and Health Technology Development Plan Project of Shandong Province (Grant No. 2019WS377), Traditional Chinese Medicine Research Project of Qingdao City (Grant No. 2020-zyy059), and Shandong Provincial Natural Science Foundation of China, Youth Project (Grant No. ZR2021QH058).

## Conflict of Interest

The authors declare that the research was conducted in the absence of any commercial or financial relationships that could be construed as a potential conflict of interest.

## Publisher's Note

All claims expressed in this article are solely those of the authors and do not necessarily represent those of their affiliated organizations, or those of the publisher, the editors and the reviewers. Any product that may be evaluated in this article, or claim that may be made by its manufacturer, is not guaranteed or endorsed by the publisher.
